# The reliability and validity of the Turkish version of the school-based asthma and allergy screening questionnaires

**DOI:** 10.1186/s12887-021-02823-9

**Published:** 2021-08-16

**Authors:** Mahmut Kilic, Ghaniya Ede, Tugba Uzuncakmak

**Affiliations:** 1 Faculty of Medicine, Department of Public Health, Yozgat Bozok University, E.Akdağ Kampüsü, 66900 Yozgat, Turkey; 2 Faculty of Medicine, Department of Pediatrics, Yozgat Bozok University, Yozgat, Turkey; 3 Faculty of Health Sciences, Department of Nursing, Yozgat Bozok University, Yozgat, Turkey

**Keywords:** Validity and Reliability, Students, Asthma, Allergy, Questionnaires

## Abstract

**Background:**

Asthma is an important public health disease affecting children that may result in school absenteeism and hospitalization. This study aims to assess the validity, reliability, and diagnostic accuracy of the Turkish version of the asthma and allergy screening questionnaire.

**Methods:**

This study included primary and secondary school students from grades 2 to 7 and their parents or caregivers. For validity, 40 children with asthma and 39 children with allergies diagnosed by the questionnaire were used to determine the sensitivity. The specificity was calculated by using the data of 100 children without asthma and allergies. The study was completed with the participation of 704 students and parents. The intraclass correlation coefficient (ICC) was used to assess item reliability. Receiver operating characteristic (ROC) analysis was used to assess validity.

**Results:**

When the cutoff point of the questionnaire was 2/3 for asthma, the sensitivity was 80.0% and 82.5% and the specificity was 56.6% and 76.8% according to the student and parent form, respectively. When the cutoff point of the questionnaire was 0/1 for allergies, the sensitivity was 74.4% and 84.6% and the specificity was 66.3% and 54.2% according to the student (SQ) and parent questionnaire (PQ), respectively. The reliability of test-retest correlation values (≥0.68) of asthma and allergy questionnaires were found to be statistically significant. The internal consistency Cronbach’s α values of the asthma SQ and PQ were 0.72 and 0.80, respectively.

**Conclusions:**

According to the Turkish questionnaire for students and their parents, the sensitivity of asthma and allergy questionnaires was similar in proportion to the original questionnaire. The Turkish version of the questionnaire can be used for asthma and allergy screening in schools.

**Supplementary Information:**

The online version contains supplementary material available at 10.1186/s12887-021-02823-9.

## Background

Asthma is a chronic disease characterized by varying degrees of airway obstruction, airway inflammation, airway hypersensitivity, wheezing, shortness of breath, and cough. Asthma causes nonmicrobial inflammation of the airways [1]. Asthma is an important public health disease affecting children and causing school absenteeism and hospitalization. In recent years, the incidence of asthma has increased, especially in children. Asthma ranks third in the 5- to 9-years-old age group in terms of years lost due to disability (YLD) [2]. Asthma symptoms are most common (>20%) in children 13- to 14-years-old in Australia, Europe, North America, and some parts of Latin America. Asthma has the highest burden on children between 5- to 14-years-old [3]. The median age at the diagnosis of asthma was 4 years. However, more than 20% of children diagnosed with asthma have symptoms of asthma in the first years of life [4]. Kuyucu et al. (2006) reported that the prevalence of allergic rhinitis in schoolchildren 9- to 11-years-old was 36.3%, and 20.4% of them were found to be positive for at least one allergen. Loss of school days, reduced academic success, and severely restricted daily activities are the most important costs for children [5, 6].

In the literature, school-based asthma screening studies using scales were reported to be a good method to identify students who were not previously diagnosed with asthma [7, 8]. In Turkey, there are not enough statistical data on the prevalence of asthma in children. Therefore, there is a need for studies in this field. The scale developed by the World Health Organization (WHO) and the International Study of Asthma and Allergies in Childhood (ISAAC) has been used to detect asthmatic students [9]. However, the application of the ISAAC scale requires video image evaluation; thus, it is a difficult scale to apply and evaluate. On the other hand, Redline et al. (2004) developed a School-Based Asthma and Allergy Screening Questionnaire in the United States. This is easy to implement and to evaluate children 7- to 13-years-old from different ethnic and socioeconomic groups. There is no video demonstration on this new screening scale. Therefore, teachers and health personnel can easily use this scale for screening asthma and allergies and evaluating its results. Within the scope of school health services, the use of a valid and reliable questionnaire by teachers and primary health care professionals can contribute to the early detection and timely treatment of children with asthma while reducing direct and indirect costs. This study aimed to investigate the validity, reliability, and diagnostic accuracy of the Turkish version of the School-Based Asthma and Allergy Screening Questionnaire.

## Methods

This is a diagnostic accuracy study. Permission for this study was obtained from the Provincial Directorate of the Ministry of Education, and ethics committee approval was obtained from the Bozok University Faculty of Medicine Ethics Committee (decision date and number: 03 June 2014 - 12/9 respectively). The students and their parents or caregivers were informed about the research, and written consent was obtained. Permission for use of the School-Based Asthma and Allergy Screening Questionnaire was obtained from the journal in which it was published [10]. The research was conducted according to the rules and ethics codes described in the Declaration of Helsinki.

### Participants

This study was carried out among students in grades 2 to 7 of primary and secondary schools in Yozgat between July 2014 and December 2015. Primary and secondary schools in the city center were divided into 3 groups according to the socioeconomic level of the region, and one primary and one secondary school were selected randomly from each group. A total of 900 students and their families were planned to be enrolled in the study, including 300 students from each region. The sample consisted of students from grades 2, 3, and 4 from three primary schools and grades 5, 6, and 7 from three middle schools (approximately 50 students and their parents from each grade). Two classes from each grade were selected randomly and included in the study. The sample size used in the diagnostic test was calculated as per receiver operating characteristic (ROC) analysis. When the area under the curve (AUC) = 0.8 and 1-β = 0.9 for power analysis, the sample size was calculated as at least n = 26 children for the case group [11]. The minimum sample size was planned to be n = 26 children with 13 cases and 13 controls and more children to be included in the sample. In this study, 40 children in the case group and 99 children in the control group had asthma. For allergies, these groups were 39 and 83, respectively. The study was completed with the participation of 704 students and parents.

### Instruments

The data were obtained from sociodemographic characteristics of students, asthma, and allergy screening questionnaires. The data consisted of sociodemographic information about children’s age, sex, grade, smoking in the family, smoking near the child indoors in the last 7 days, parents' education status, type of house heating, family members with asthma, family income, etc. The school-based asthma and allergy screening questionnaire consisted of two forms: the student questionnaire form (SQ), which consisted of 13 questions, and the parent or caregiver questionnaire form (PQ), which consisted of 14 questions (Additional file 1). In the SQ, questions 1 to 7 were related to asthma, and questions 8 to 9 were related to allergies. In the PQ, questions 1 to 8 were related to asthma, and questions 9 to 10 were allergy-related. In the asthma and allergy questionnaires, the answers given to these questions were scored as ‘0’ for never, ‘1’ for occasionally, and ‘2’ often. When calculating the questionnaire total score, 1 point was given if one of the answers was marked sometimes or a lot. In the SQ, for asthma, the total score was 7, and for allergies, it was 2. In the PQ, for asthma, the total score was 8, and for allergies, it was 2. The last 4 questions in both forms were about the diagnosis of asthma and allergies, and the use of drugs was not scored [10]. In addition, 4 questions were added to the SQ and 7 questions were added to the PQ regarding sociodemographic characteristics.

In the original validity study of the SQ, for asthma, the sensitivity was 80% and the specificity was 70% for those who answered at least 3 of the 7 asthma-related questions as occasionally or often (cutoff point ≥3). According to the PQ, for asthma, the sensitivity was 58%, and the specificity was 69% for those who answered at least 2 of the 8 asthma-related questions as occasionally or often (cutoff point ≥2). For allergies, the sensitivity of the SQ and PQ were 81% and 78%, and the specificity was 42% and 53%, respectively, for those who answered one of two questions related to allergies as occasionally or often (cutoff point ≥1) [10].

### Procedure

#### Turkish translation of the questionnaire

First, the questionnaire was translated from English to Turkish by two English lecturers. The questionnaire was then assessed by two experts, one chest disease specialist and one pediatric specialist, who then developed a Turkish version of the questionnaire. Then, the Turkish version was translated back to English by a bilingual person. The linguistic validation of the questionnaires was completed by 2 linguistic experts following the opinions of the students and their families. The original and translated questionnaires were compared to determine whether the translated version conveyed the same meaning as the original. The translated questionnaire was determined to be sufficient, and 6 students and 6 parents (sex and age balanced) were interviewed separately for the intelligibility of the Turkish version of the questionnaire. Finally, the final Turkish version of the questionnaire was formed.

### Data collection

The researchers explained the study’s purpose to the students. The students took the informed consent form and PQ form home, and their parents/caregivers completed them. On the following day, the informed consent form and PQ were collected from the students. The SQ form was given to the students after an explanation from the researchers. The students were instructed to mark the most suitable option.

### Reliability of the questionnaire

For the test-retest reliability, one class was randomly selected from each grade, grades 2 to 7, participating in the research. These students and their parents/caregivers (200) were readministered the same questionnaires one week later. The questionnaire was repeated with 154 students and 111 parents.

### The validity of the questionnaire

The sensitivity and specificity of the questionnaire were measured to assess validity. The SQ (items 10 to 13) and PQ (items 11 to 14) are relevant to diagnosing asthma and allergies. To identify the sensitivity of the questionnaire, students who were previously diagnosed with asthma or allergy by a physician and according to the PQ results and students who had a risk for asthma or allergy were referred to the Child Health and Diseases policlinic of Bozok University Research and Application Hospital. The parents who reported that their children were diagnosed with asthma or took asthma medication were interviewed. The children whose diagnostic procedures were considered suspicious were re-evaluated in the hospital, and those with definite disease were identified. The same procedures were used for allergies. Of the referred students, 40 were diagnosed with definite asthma by anamnesis, clinical examination, and pulmonary function test (PFT) results. The anamnesis, clinical examination, and skin prick test confirmed the definitive diagnosis of allergy in 39 students.

In the PQ, the parents of children who were not diagnosed with asthma or allergies were interviewed. The parents were asked to take their children to the hospital for control of asthma and allergies. The anamnesis, clinical examination, and PFT results were reviewed by the pediatrician, and asthma was ruled out in 99 students. The same physician confirmed that 83 students were free of allergies according to anamnesis, clinical examination, and skin prick tests. The sensitivity of the questionnaire was determined according to the patients with definite asthma or allergy diagnosis, and the specificity of the questionnaire was compared to those without definite asthma or allergy diagnosis.

### Data analysis

The data were analyzed in IBM SPSS Statistics Standard Concurrent User V 25, Authorization Code: e31d836848b0a60e5756. The Cronbach's α coefficient for the internal consistency of the questionnaire and the test-retest results of the items for reliability were analyzed using the intraclass correlation coefficient (ICC) test. The calculation of the sensitivity and specificity of various cutoff values of SQ and PQ for predicting asthma and allergies were performed by ROC analysis.

## Results

**Fig. 1:**
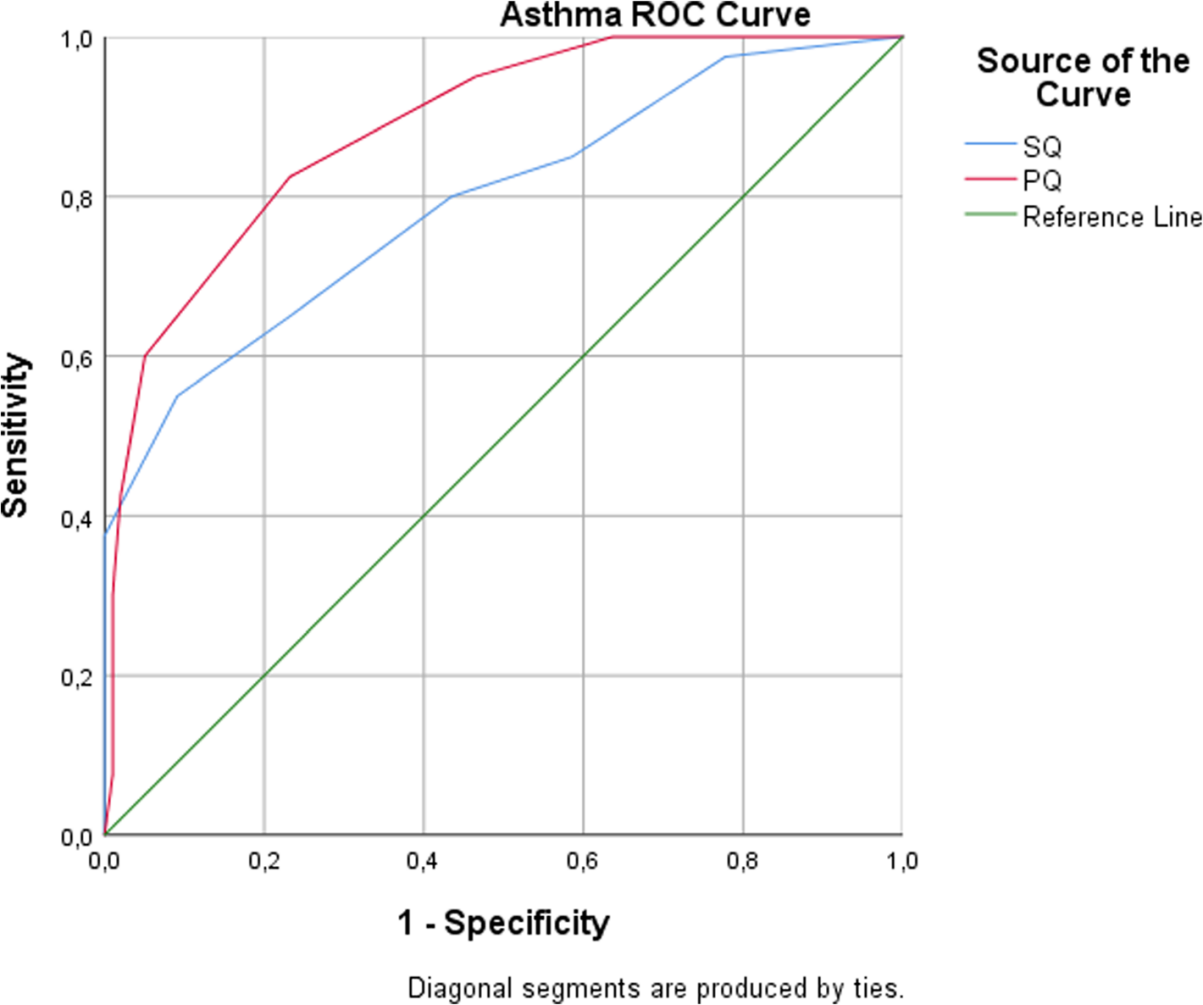
Area under the ROC curve for asthma questionnaires

The SQ and PQ questionnaires used a cutoff score of ≥3 to predict asthma. The sensitivity of the SQ was 80% (true positive (32)/diagnosed with asthma (40) x 100), and the specificity was 56.6% (true negative (56)/asthma absent (99) x 100). The sensitivity of the PQ was 82.5% (true positive (33)/diagnosed with asthma (40) x 100), and the specificity was 76.8% (true negative (76)/asthma absent (99) x 100) (Table 1). The area under the ROC curve for the asthma questionnaire was 0.793 for the SQ and 0.886 for the PQ (Fig. 1, Table 2).

**Table 1:** Validity of asthma and allergy questionnaires

**Asthma screening questionnaire**	**Asthma reference test (Physician-diagnosis + PFT)**		
**Asthma SQ** (cut of point 2/3)	**Asthma diagnosed**	**Not asthma**	**Total**
**Asthma suspect**	**True positive (32)**	False positive (43)	**84**
**Not asthma**	False negative (8)	**True negative (56)**	**55**
**Total**	**40**	**99**	**139**
	**Sensitivity 80.0%**	**Specificity 56.6%**	
**Asthma PQ** (cut of point 2/3)			
**Asthma suspect**	**True positive (33)**	False positive (23)	**84**
**Not asthma**	False negative (7)	**True negative (76)**	**55**
**Total**	**40**	**99**	**139 (100.0)**
	**Sensitivity 82.5%**	**Specificity 76.8%**	
**Allergy screening questionnaire**	**Allergy Reference test (Physician-diagnosis +Prick test)**		
**Allergy SQ**	**Allergy diagnosed**	**Not allergy**	**Total**
**Allergy suspect**	**True positive (29)**	False positive (28)	**57**
**Not allergy**	False negative (10)	**True negative (55)**	**65**
**Total**	**39**	**83**	**122**
	**Sensitivity 74.4%**	**Specificity 66.3%**	
**Allergy PQ**			
**Allergy suspect**	**True positive (33)**	False positive (38)	**71**
**Not allergy**	False negative (6)	**True negative (45)**	**51**
**Total**	**39**	**83**	**122**
	**Sensitivity 84.6%**	**Specificity 54.2%**	

*SQ* Student Questionnaire, *PQ* Parent / Guardian Questionnaire

**Table 2:** The area under the ROC curve for asthma and allergy questionnaires

Test result variables	Area	95% Confidence Interval		Sig. (2-sided)
		Lower Bound	Upper Bound	
**Asthma**				
Asthma SQ	0.793	0.705	0.881	0.000
Asthma PQ	0.886	0.828	0.944	0.000
**Allergy**				
Allergy SQ	0.711	0.612	0.810	0.000
Allergy PQ	0.740	0.646	0.834	0.000

*SQ* Student Questionnaire, *PQ* Parent / Guardian Questionnaire

**Fig. 2:**
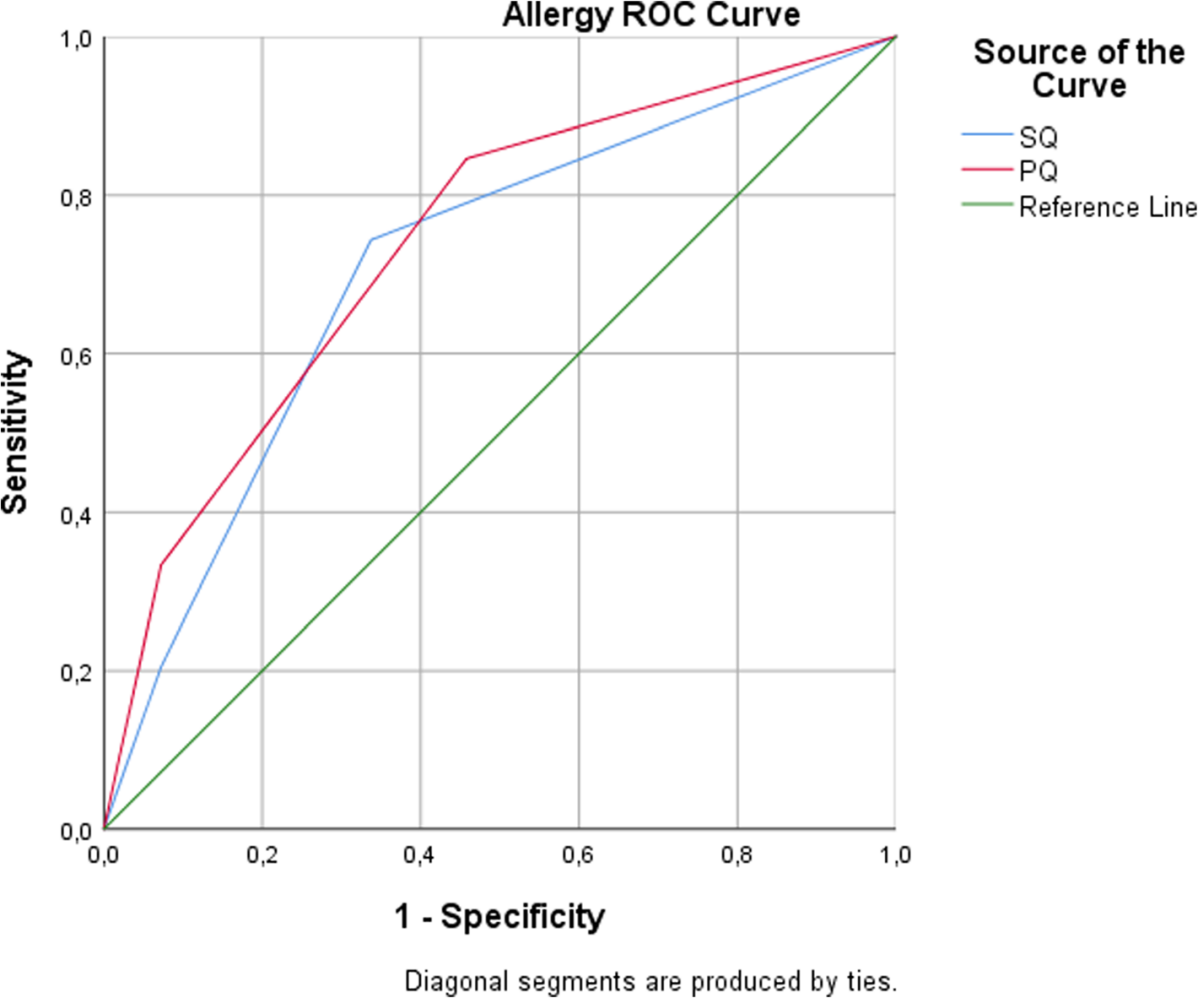
Area under the ROC curve for allergy questionnaires

When the cutoff point for the allergy questionnaires was taken as ≥1 according to the ROC analysis, the sensitivity of the SQ was 74.4% (true positive (29)/allergy diagnosed (39) x 100), and the specificity was 66.3% (true negative (55)/allergy absent (83) x 100). According to the PQ, the sensitivity of the questionnaire was calculated as 84.6% (true positive (33)/allergy diagnosed (39) x 100), and the specificity was 54.2% (true negative (45)/allergy absent (83) x 100) (Table 1). The area of the allergy questionnaire under the ROC curve was 0.711 in the SQ and 0.740 in the PQ (Fig. 2, Table 2).

For the reliability of the asthma questionnaire, when the correlation between test-retest total scores was examined, it was found that r = 0.68 in the SQ and r = 0.75 in the PQ. The correlation coefficient of test-retest total scores of the allergy questionnaire was found to be r = 0.68 in both the SQ and PQ. Test-retest correlation values of both questionnaires were found to be statistically significant (Table 3). The internal consistency Cronbach’s α values of the asthma SQ and PQ were 0.72 and 0.80, respectively. In factor analysis, asthma SQ and PQ were both found to have one factor (Table 4). For asthma SQ and PQ, the Kaiser-Meyer-Olkin (KMO) values were 0.827 and 0.875, respectively, the Bartlett test of sphericity was significant at p<0.0001, and the variance of extraction sums of squared loadings were 38.379% and 44.418%, respectively.

**Table 3:** The test-retest reliability, computed as an intra-class correlation coefficient

Asthma SQ	Intraclass Correlation	95% Confidence Interval		F Test with True Value 0		
		Lower Bound	Upper Bound	Value	df	Sig
Single Measures	0.682	0.588	0.758	5.297	153	0.000
Average Measures	0.811	0.741	0.863	5.297	153	0.000
**Asthma PQ**						
Single Measures	0.722	0.620	0.801	6.206	110	0.000
Average Measures	0.839	0.765	0.889	6.206	110	0.000
**Allergy SQ**						
Single Measures	0.671	0.574	0.750	5.085	153	0.000
Average Measures	0.803	0.730	0.857	5.085	153	0.000
**Allergy PQ**						
Single Measures	0.712	0.607	0.793	5.951	110	0.000
Average Measures	0.832	0.755	0.885	5.951	110	0.000

Two-way mixed effects model where people effects are random and measures effects are fixed

*SQ* Student Questionnaire, *PQ* Parent / Guardian Questionnaire

**Table 4:** Asthma questionnaire’s reliability analysis, item-total statistics

Asthma SQ	Scale Mean if Item Deleted	Scale Variance if Item Deleted	Corrected Item-Total Correlation	Cronbach’s Alpha if Item Deleted
Item 1	2.81	5.264	0.451	0.686
Item 2	2.78	5.309	0.374	0.701
Item 3	2.51	4.844	0.449	0.684
Item 4	2.21	4.710	0.380	0.710
Item 5	2.79	5.047	0.485	0.677
Item 6	2.90	5.328	0.442	0.689
Item 7	2.58	4.799	0.493	0.672
**Asthma PQ**				
Item 1	2.09	5.769	0.593	0.774
Item 2	2.06	5.602	0.576	0.774
Item 3	1.89	5.379	0.533	0.781
Item 4	1.77	5.498	0.395	0.811
Item 5	2.00	5.526	0.580	0.773
Item 6	2.17	6.022	0.558	0.781
Item 7	1.95	5.363	0.584	0.772
Item 8	2.18	6.299	0.441	0.795

Cronbach α value of the asthma SQ (student questionnaire) and PQ (parent / guardian questionnaire) were 0.721 and 0.805 respectively

## Discussion

In this study, the sensitivity of the Turkish version of the SQ for allergies (74.4%) was lower than that of the original questionnaire (81%), while the specificity (66.3%) was significantly higher than that of the original questionnaire (42%). The sensitivity of the Turkish version of the allergy PQ (84.6%) was higher than the sensitivity of the original questionnaire (78%), and the specificity of the Turkish version (54.2%) and the specificity of the original questionnaire (53%) were similar (Tables 1 and 5) [10]. For validity, in the Turkish version, the sensitivity of the allergy SQ was lower and the specificity was higher than that of the original questionnaire. The sensitivity rate of the Turkish allergy SQ may have been lower than that of the original scale because Turkish children may be less aware of allergy symptoms than U.S. children. The sensitivity and specificity of the Turkish version of the allergy PQ were higher than those of the original questionnaire. This may be due to Turkish women working less than U.S. women; therefore, they may spend more time with their children, and consequently, mothers may have a better opportunity to observe their children. In a study using the International Childhood Asthma Allergy Questionnaire, the sensitivity was found to be 74% [12]. In a study in which the questionnaire was translated into Turkish, the prevalence of asthma, wheezing, and rhinitis was 14.1%, 22.4%, and 12.9%, respectively [13].

**Table 5:** ROC Coordinates of the curve for asthma and allergy questionnaires

Test Result Variable(s) Asthma questionnaires	Cutoff Value	Sensitivity	1 – Specificity
Asthma Student Questionnaire	-1.00	1.000	1.000
	0.50	0.975	0.778
	1.50	0.850	0.586
	**2.50**	**0.800**	**0.434**
	3.50	0.650	0.232
	4.50	0.550	0.091
Asthma Parent/Guardian Questionnaire	-1.00	1.000	1.000
	0.50	1.000	0.636
	1.50	0.950	0.465
	**2.50**	**0.825**	**0.232**
	3.50	0.700	0.131
	4.50	0.600	0.051
**Allergy questionnaires**			
Allergy Student Questionnaire	-1.00	1.000	1.000
	**0.50**	**0.744**	**0.337**
	1.50	0.205	0.072
	3.00	0.000	0.000
Allergy Parent/Guardian Questionnaire	-1.00	1.000	1.000
	**0.50**	**0.846**	**0.458**
	1.50	0.333	0.072
	3.00	0.000	0.000

For asthma, the sensitivity of the Turkish version of the SQ (80%) and the sensitivity of the original SQ (80%) were the same, while the specificity was lower in the Turkish version (56.6%) than in the original version (70%). The Turkish asthma SQ specificity rate was lower than that of the original scale, which may be because nonasthmatic children stated the presence of symptoms more often on the 3 items of the scale (It is hard for me to stop coughing, 43.4%; My chest feels tight or hurts after I run, play hard, or do sports, 58.6%; I cough when I run, climb stairs, or play sports, 42.4%). For asthma, the sensitivity (82.5%) and specificity (76.8%) of the Turkish version of the PQ were higher than the sensitivity (58%) and specificity (69%) of the original version of the PQ (Table 1). The cutoff point for asthma was taken as ≥2, which is the same as the original PQ. The Turkish version of the PQ had very high sensitivity (95%) compared to the original (58%), and its specificity (53.5%) was lower than that of the original (69%). Therefore, taking ≥3 as the cutoff point in the asthma PQ provides a more appropriate level of sensitivity and specificity (Table 5) [10]. In the Turkish validity study, the sensitivity of the asthma SQ was the same as that of the original, but its specificity was lower than that of the original. The sensitivity and specificity levels of the asthma PQ were higher than those of the original questionnaire. The sensitivity rate of the Turkish asthma PQ was higher than that of the original scale, which may be because Turkish women work less; therefore, they take care of their children, and consequently, mothers have a better opportunity to observe their children.

Validity and reliability studies of the questionnaires used for asthma in children can be found in the literature. In Turkey, for children 4- to 11-years-old, childhood asthma control test sensitivity was 74.8% and specificity was 88.7% [14]. The sensitivity of a questionnaire used to identify children and adolescents with asthma in Brazil was found to be 74% [7]. The sensitivity of the questionnaire used in asthma screening in children 5- to 15-years-old was found to be 70% [8]. The sensitivity and specificity of the asthma questionnaire administered to preschool children in Latin America were found to be 93.1% [15]. In the United States, the sensitivity and specificity of asthma questionnaires applied to children in grades 3 to 5 of primary school were 90% and 49%, respectively [16]. In a study in which primary school children were followed up with an asthma questionnaire for 2 years, the sensitivity and specificity of the questionnaire were 94% and 87% in the first year and 96% in the second year [17]. In a study using the European Respiratory Health Questionnaire, the sensitivity and specificity of the questionnaire were found to be 75.1% and 80.1%, respectively [18]. In another study, the sensitivity and specificity of the asthma control questionnaire applied to asthma patients were 78% and 77.5%, respectively [19]. In the asthma screening survey applied to children (9- to 12-years-old) and their families admitted to a hospital in Argentina, it was detected that the sensitivity of the student version (cutoff point ≥2) was 53.4% and the specificity was 84.3% and the sensitivity of the parent version (cutoff point ≥3) was 92.3% and the specificity was 86.4% [20].

In this study, the internal consistency (0.72, 0.80) of the asthma student and parent/caregiver forms was adequate. Turkey's childhood asthma control test reliability (test-retest) was 0.71. The internal consistency Cronbach’s α value was found to be 0.69 for children, and for the parents, the value was determined to be 0.78 [14]. In the language validity and reliability study of the Asthma Screening Scale in Argentina, the internal consistency Cronbach’s α value of the 9- to 12-year-old student questionnaire was found to be 0.69, and that of the parental questionnaire value was 0.88 [20]. Since there were only two allergy questions, internal consistency was not considered. The reliability of the Turkish version of the School-Based Asthma and Allergy Screening Questionnaire was satisfactory according to the test-retest result (r = 0.68). Therefore, the questionnaire had reliable characteristics (Table 3). Asthma SQ and PQ were both found to have one-factor loadings in the factor analysis. In this study, Turkish primary health care professionals and teachers gained a screening questionnaire that is easy to implement and evaluate for asthma and allergies in primary and secondary school students. In Turkey, children benefit from health services free of charge. Every family has a family doctor. Family physicians are obliged to follow up with their registered patients once a year. Therefore, the examination of false-positive cases by health institutions does not create a burden on the health system.

## Conclusions

The Turkish version of the School-Based Asthma and Allergy Screening Questionnaire had a similar level of sensitivity and specificity as the original asthma and allergy screening questionnaires used in the literature. The reliability of the Turkish version of the questionnaire was acceptable. The Turkish version of the questionnaire can be used to screen for asthma and allergies in students in grades 2 to 7 in Turkey.

## Supplementary Information

**Additional file 1.**

## Data Availability

The study data are stored. The data may be provided if desired. The datasets used and analyzed during the current study are available from the corresponding author on reasonable request.

## Abbreviations

YLD: Years lost due to disability
WHO: World Health Organization
ISAAC: International Study of Asthma and Allergies in Childhood
SQ: Student questionnaire
PQ: parent or caregiver questionnaire
PFT: Pulmonary function test
SPSS: Statistical Package for the Social Sciences
ICC: Intraclass correlation coefficient
ROC: Receiver operating characteristics
KMO: Kaiser-Meyer-Olkin
