# Supplementary material for: The reliability and validity of the Turkish version of the school-based asthma and allergy screening questionnaires
Source: BMC Pediatr. 2021 Aug 16;21:348. doi: 10.1186/s12887-021-02823-9 (PMC8365918; doi:10.1186/s12887-021-02823-9)
Supplement: Supplementary file 1 — Additional file 1. [file 12887_2021_2823_MOESM1_ESM.docx]

# The reliability and validity of the Turkish version of the school-based asthma and allergy screening questionnaire

**ÖĞRENCİ ANKETİ**

Okul adı: ……………………………………….. Sınıf: ………………….

Ad ve soyad : …………………..……………… Okul No: …………….

Yaş: ……….. Cinsiyet: 1)Erkek, 2) Kız .

| **Aşağıdaki durumları ne sıklıkta yaşarsın? Lütfen sana uygun olanı işaretle.** | | | |
| --- | --- | --- | --- |
| 1. Nefes alıp verirken göğsümden hırıltı veya gürültü sesi gelir. | □ Hiçbir zaman | □ Ara sıra | □ Çoğu kez |
| 1. Derin nefes almakta zorlanırım. | □ Hiçbir zaman | □ Ara sıra | □ Çoğu kez |
| 1. Öksürüğümü durdurmakta zorlanırım. | □ Hiçbir zaman | □ Ara sıra | □ Çoğu kez |
| 1. Koştuktan, yorucu bir oyundan veya spordan sonra göğsümde ağrı ya da sıkışma, kasılma hissederim. | □ Hiçbir zaman | □ Ara sıra | □ Çoğu kez |
| 1. Geceleri veya sabahları öksürükle uyanırım. | □ Hiçbir zaman | □ Ara sıra | □ Çoğu kez |
| 1. Geceleri solunum sıkıntısından dolayı uyanırım. | □ Hiçbir zaman | □ Ara sıra | □ Çoğu kez |
| 1. Koşarken, merdiven çıkarken veya spor yaparken öksürürüm. | □ Hiçbir zaman | □ Ara sıra | □ Çoğu kez |
| 1. Gözlerimde kaşıntı, şişme veya yanma olur. | □ Hiçbir zaman | □ Ara sıra | □ Çoğu kez |
| 1. Burun akıntısı veya burun tıkanıklığı problemim var. | □ Hiçbir zaman | □ Ara sıra | □ Çoğu kez |

| **Lütfen aşağıdaki soruların cevaplarından sana uygun olanı işaretle.** |  |  |
| --- | --- | --- |
| 10. Doktor ya da hemşire bende astım olduğunu söyledi. | □Evet | □Hayır |
| 11. Son bir yıl içinde solunum sıkıntısı ya da astım yüzünden gece hastanede yattım. | □Evet | □Hayır |
| 12. Astım için ilaç alırım ya da ağızıma sprey (fıs fıs) sıkarım. | □Evet | □Hayır |
| 13. Alerji için ilaç alırım. | □Evet | □Hayır |

**EBEVENY/ BAKICI ANKETİ**

Okul adı: …………………………………………………… . Sınıf: ………………….

Öğrencinin Adı ve soyadı : …………………..……………… Okul No: …………….

| **Çocuğunuz aşağıdaki durumları ne sıklıkta yaşar?**  **Lütfen, çocuğunuzun yaşadığı sorunlara uygun olanı işaretleyiniz.** | | | | |
| --- | --- | --- | --- | --- |
| 1. Çocuğunuz nefes alıp verirken göğsünden hırıltı veya gürültü sesi gelir mi? | □ Hiçbir zaman | □ Ara sıra | □ Çoğu kez | □ Bilmiyorum |
| 1. Çocuğunuz derin nefes alırken zorlanır mı? | □ Hiçbir zaman | □ Ara sıra | □ Çoğu kez | □ Bilmiyorum |
| 1. Çocuğunuz öksürüğünü durdurmakta zorlanır mı? | □ Hiçbir zaman | □ Ara sıra | □ Çoğu kez | □ Bilmiyorum |
| 1. Çocuğunuz koştuktan, yorucu bir oyundan veya spordan sonra göğsünde ağrı ya da sıkışma, kasılma hisseder mi? | □ Hiçbir zaman | □ Ara sıra | □ Çoğu kez | □ Bilmiyorum |
| 1. Çocuğunuz geceleri öksürük nedeniyle uyanır mı? | □ Hiçbir zaman | □ Ara sıra | □ Çoğu kez | □ Bilmiyorum |
| 1. Çocuğunuz geceleri solunum sıkıntısından dolayı uyanır mı? | □ Hiçbir zaman | □ Ara sıra | □ Çoğu kez | □ Bilmiyorum |
| 1. Çocuğunuz koşarken, merdiven çıkarken veya spor yaparken öksürür mü? | □ Hiçbir zaman | □ Ara sıra | □ Çoğu kez | □ Bilmiyorum |
| 1. Çocuğunuzun solunum problemleri nedeniyle okula gidemediği günler oldu mu? | □ Hiçbir zaman | □ Ara sıra | □ Çoğu kez | □ Bilmiyorum |
| 1. Çocuğunuzun gözlerinde kaşıntı, şişme veya yanma olur mu? | □ Hiçbir zaman | □ Ara sıra | □ Çoğu kez | □ Bilmiyorum |
| 1. Çocuğunuzda burun tıkanıklığı veya burun akıntısı problemi var mı? | □ Hiçbir zaman | □ Ara sıra | □ Çoğu kez | □ Bilmiyorum |

| **Lütfen, çocuğunuzla ilgili aşağıdaki sorulara uygun olan cevabı işaretleyiniz.** | | | |
| --- | --- | --- | --- |
| 11. Doktor ya da hemşire çocuğunuzda astım, aktif bir solunum yolu hastalığı veya hırıltılı bronşiti olduğunu söyledi mi? | □Evet | □Hayır | □ Bilmiyorum |
| 12. Çocuğunuz son bir yıl içinde solunum sıkıntısı ya da astım nedeniyle gece hastanede yattı mı? | □Evet | □Hayır | □ Bilmiyorum |
| 13. Çocuğunuz astım için ilaç alır mı veya ağızına sprey (fıs fıs) sıkar mı? | □Evet | □Hayır | □ Bilmiyorum |
| 14. Çocuğunuz alerji için ilaç alır mı? | □Evet | □Hayır | □ Bilmiyorum |

**STUDENT QUESTIONNAIRE**

**School name: …………………………………………. Grade: …………….**

**Name and surname: ……………………………… Student ID: …………….**

**Age: ………… .. Sex:** 1) Boy 2) Girl

| **Please tell us how often you have any of the following:** | | Never | Sometimes | A lot |
| --- | --- | --- | --- | --- |
| 1. | My breathing sounds noisy or wheezy. |  |  |  |
| 2. | It is hard to take a deep breath. |  |  |  |
| 3. | It is hard for me to stop coughing. |  |  |  |
| 4. | My chest feels tight or hurts after I run, play hard, or do sports. |  |  |  |
| 5. | I wake up at night coughing. |  |  |  |
| 6. | I wake up at night because I have trouble breathing. |  |  |  |
| 7. | I cough when I run, climb stairs or play sports. |  |  |  |
| 8. | My eyes get itchy, puffy or burn. |  |  |  |
| 9. | I have problems with a runny or stuffy nose. |  |  |  |
| **Please answer the following questions:** | | | Yes | No |
| 10. | A doctor or nurse told me that I have asthma. | |  |  |
| 11. | I stayed in the hospital overnight for asthma or trouble breathing this past year. | |  |  |
| 12. | I take medicine or use an inhaler for asthma. | |  |  |
| 13. | I take medicine for allergies. | |  |  |

**PARENT OR GUARDIAN QUESTIONNAIRE**

**School name: …………………………………………. Grade: …………….**

**Child's name and surname: ………………………… Student ID: …………….**

| Please tell us how often your child has any of the following. (If your child has more problems in some seasons of the year, please tell us about problems during the *worst* season.) Does your child . . . | | | | | |
| --- | --- | --- | --- | --- | --- |
|  |  | Never | Sometimes | A lot | Don't know |
| 1. | Make noisy or wheezy sounds when breathing? |  |  |  |  |
| 2. | Have a hard time taking a deep breath? |  |  |  |  |
| 3. | Develop coughs that won't go away? |  |  |  |  |
| 4. | Complain about a chest that feels tight or hurts after running, playing hard, or doing sports? |  |  |  |  |
| 5. | Wake up at night coughing? |  |  |  |  |
| 6. | Wake up at night because of trouble breathing? |  |  |  |  |
| 7. | Cough when running, climbing stairs or playing sports? |  |  |  |  |
| 8. | Miss days of school (absent from school) because of breathing problems? |  |  |  |  |
| 9. | Have eyes that itch, get puffy or burn. |  |  |  |  |
| 10. | Have problems with a runny, stuffy nose. |  |  |  |  |
| **Please answer the following questions about your child:** | | | Yes | No | Don't Know |
| 11. | Has a doctor or nurse told you that your child has asthma, reactive airway disease or wheezy bronchitis? | |  |  |  |
| 12. | Has your child stayed in the hospital overnight for asthma or for trouble breathing this past year? | |  |  |  |
| 13. | Does your child take medicine (or use an inhaler) for asthma? | |  |  |  |
| 14. | Does your child take medicine for allergies? | |  |  |  |
